# Body Mass Index Modulates the Impact of Short-Term Exposure to Air Particulate Matter on High-Density Lipoprotein Function

**DOI:** 10.3390/antiox11101938

**Published:** 2022-09-28

**Authors:** Alice Ossoli, Chiara Favero, Luisella Vigna, Angela Cecilia Pesatori, Valentina Bollati, Monica Gomaraschi

**Affiliations:** 1 Center E. Grossi Paoletti, Department of Pharmacological and Biomolecular Sciences, Università degli Studi di Milano, 20133 Milan, Italy; 2 EPIGET Lab, Department of Clinical Sciences and Community Health, Università degli Studi di Milano, 20122 Milan, Italy; 3 Fondazione IRCCS Ca’ Granda Ospedale Maggiore Policlinico, 20122 Milan, Italy

**Keywords:** high-density lipoproteins, air pollution, endothelium, nitric oxide, body mass index

## Abstract

Air particulate matter (PM) exposure has been associated with increased cardiovascular risk, especially in obesity. By triggering inflammation and oxidative stress, PM could impact atheroprotection by high-density lipoproteins (HDL). The aim of the study was to assess the relationship between short-term exposure to PM and HDL function, and the modifying effect of body mass index (BMI). Daily exposures to PM_10_ and PM_2.5_ of 50 subjects with overweight/obesity and 41 healthy volunteers with BMI < 30 kg/m^2^ were obtained from fixed monitoring stations. HDL function was assessed as promotion of nitric oxide (NO) release by endothelial cells and reduction in cholesterol in macrophages. HDL-induced NO release progressively declined with the increase in BMI. No association was found between HDL function and PM exposure, but a modifying effect of BMI was observed. The positive association between PM_10_ exposure at day −1 and NO production found at normal BMI values was lost in participants with higher BMI. Similar results were obtained for the reduction in macrophage cholesterol. The loss of the compensatory response of HDL function to PM exposure at increasing BMI levels could contribute to the endothelial dysfunction induced by PM and help to explain the susceptibility of subjects with obesity to air pollution.

## 1. Introduction

Ischemic heart disease (IHD) and stroke are the main causes of morbidity and mortality worldwide [1], with hypertension, diabetes, obesity, and dyslipidemia representing the most important modifiable risk factors. Regarding dyslipidemia, higher plasma levels of total and non-high-density lipoprotein cholesterol and reduced high-density lipoprotein cholesterol (HDL-C) are associated with an increased risk of IHD and stroke [2]. Nowadays, both short- and long-term exposures to environmental pollution are also recognized as relevant risk factors for cardio- and cerebrovascular diseases [1]. The detrimental effect of pollution is possibly related to the ability of air pollutants to trigger inflammation and oxidative stress, which can directly affect the cardiovascular system or indirectly impair other key players such as blood pressure and lipids [3]. There is evidence for increased susceptibility to the effects of pollution for elderly subjects, those with obesity, or in secondary prevention [4].

Exposure to air pollutants has been variably associated with changes in plasma lipid profile, including a reduction in HDL-C [5]. HDL can exert a series of protective activities against the development and progression of atherosclerosis [6]. The key role of HDL in the reverse cholesterol transport (RCT) from peripheral cells, especially macrophages in the arterial wall, to the liver has been widely considered the most relevant atheroprotective function of HDL. In particular, HDL are the main extracellular acceptors of cholesterol from cells, which represents the first and rate-limiting step of RCT. Moreover, HDL can exert antioxidant and anti-inflammatory activities and can preserve endothelial homeostasis. Since endothelial dysfunction is deeply involved in atherosclerosis development and progression, the ability of HDL to preserve the endothelial barrier as a continuous and anti-adhesive layer and to contribute to the regulation of vascular tone has gained attention. In particular, the capacity of HDL to promote the release of nitric oxide (NO) by endothelial cells was tested in the context of several clinical conditions [7]. The development of cell-free and cell-based assays to measure the various atheroprotective activities of HDL allowed understanding that many pathologic conditions, as well as therapeutic interventions, can affect HDL function independently from changes in HDL-C, suggesting that HDL function is more relevant than their concentrations (at least when expressed as their cholesterol content) to estimate HDL-mediated cardiovascular protection. In particular, pathologic conditions associated with acute or chronic inflammatory states, such as obesity, diabetes, myocardial infarction, infections, and autoimmune diseases, were associated with a general impairment of HDL function [7]. In this context, since air pollutants trigger inflammation, exposure to environmental pollution could also impact HDL function. To date, the ability of HDL to promote cholesterol efflux and their antioxidant/anti-inflammatory index through a cell-free assay have been investigated in the context of short- or long-term exposure to air pollutants with inconsistent results [8]. On the contrary, the capacity of HDL to promote NO release by endothelial cells has not been investigated yet. Interestingly, it has been shown that acute exposure to diesel exhaust can impair vascular function for the following 24 h, likely due to the reduced availability of NO [9].

Thus, the aim of the present study was (i) to assess the relationship between short-term exposure to air pollutants and HDL function, measured as their ability to reduce cell cholesterol content in macrophages and to promote NO release by endothelial cells, and (ii) to investigate whether body mass index (BMI) could modulate such a relationship.

## 2. Materials and Methods

### 2.1. Subjects

Fifty subjects with overweight/obesity were enrolled at the Center for Obesity and Work (COW; Department of Preventive Medicine, IRCCS Fondazione Ca’ Granda–Ospedale Maggiore Policlinico, Milan, Italy) as part of the cross-sectional study SPHERE [10]. Forty-one healthy volunteers with BMI ≤ 30 kg/m^2^ were enrolled by answering an ad hoc developed announcement posted on the SPHERE Project website (http://users.unimi.it/sphere, accessed on 29 August 2022). Subjects were enrolled between January 2013 and March 2015. Each participant signed an informed consent form, which had been approved by the Ethics Committee of Fondazione IRCCS Cà Granda Ospedale Maggiore Policlinico (approval number 1425), in accordance with the principles of the Declaration of Helsinki [11].

### 2.2. Collection of Personal Data and Biological Samples

Epidemiological and clinical data were collected as described [10]. Blood samples were collected after an overnight fast. Serum was prepared by low-speed centrifugation at 4 °C and immediately frozen at −80 °C. Apolipoprotein B-depleted (apoB-D) serum was obtained by the precipitation of apoB-containing lipoproteins with PEG 6000 in 10 mM HEPES at 4 °C for 30 min, followed by centrifugation at 2200 g for 30 min.

### 2.3. Exposure Assessment

Short-term particulate matter (PM) with an aerodynamic diameter of 10 μm or less (PM_10_) and PM with an aerodynamic diameter of 2.5 μm or less (PM_2.5_) exposure was evaluated as one-week lag exposure time window.

Daily PM concentrations were collected from fixed monitoring stations of the Regional Environmental Protection Agency (ARPA Lombardy). Using ArcGIS^®^ software (Desktop: Release 10.1, Environmental Systems Research Institute, Esri, Redlands, CA, USA), we assigned each subject the daily PM concentrations from the nearest monitor to that subject’s home address for the six days preceding recruitment and the nearest monitor to the hospital (Ospedale Maggiore Policlinico, Milan, Italy) for the day of recruitment. Meteorological data were obtained from the ARPA monitoring stations, measuring temperature and relative humidity. The apparent temperature was calculated as previously reported [12]. Detailed information on the exposure assessment method has been previously described [13,14].

### 2.4. Biochemical Analysis

The concentrations of total cholesterol, triglycerides, and HDL-C levels in serum were assessed by standard enzymatic or turbidimetric techniques on a Cobas c311 auto-analyzer (Roche Diagnostics, Milan, Italy). Low-density lipoprotein cholesterol (LDL-C) was calculated by the Friedewald formula [15]. Inflammatory markers and mitochondrial DNA copy number (mtDNAcn) were measured as described [16].

### 2.5. Nitric Oxide Production

Primary cultures of human umbilical vein endothelial cells (HUVECs) were purchased from PromoCell (Heidelberg, Germany) and subcultured for 1–3 passages according to manufacturer instructions. Experiments were performed in M199 with 0.75% bovine serum albumin (BSA) and 1% fetal calf serum (FCS) (Euroclone, Milan, Italy). To evaluate NO production, HUVECs were incubated with apoB-D sera for 30 min. The generated NO was measured by fluorescence using a diacetate derivative of 4,5-diaminofluorescein (DAF-2 DA, Sigma-Aldrich Chemie, Steinheim, Germany) [17]. For each sample, fluorescence was normalized by the protein concentration of the total cell lysate, assessed by the microBCA method (Thermo Fischer Scientific, Waltham, MA, USA).

### 2.6. Macrophage Cholesterol Mass

The human monocytic leukaemia cell line THP-1 was purchased from the American Type Culture Collection (ATCC, Manassas, VA, USA) and cultured in Roswell Park Memorial Institute (RPMI) 1640 medium supplemented with 10% (*v*/*v*) fetal bovine serum (FBS), 1 mM sodium pyruvate, and 50 μM 2-mercaptoethanol. To induce the differentiation into macrophage-like cells, THP-1 cells were cultured in the presence of 100 ng/mL phorbol 12-myristate 13-acetate (PMA) for 72 h. Macrophages were loaded with acetylated LDL (50 μg of protein/mL) for 24 h in the presence of 2 μg/mL of an acyl-CoA:cholesterol acyltransferase inhibitor and then incubated with 2.5% apoB-D serum for 7 h. At the end of the experiment, cells were washed with PBS and lysed overnight in 1% sodium cholate and 10 U/mL of DNase (Sigma-Aldrich Chemie, Steinheim, Germany) at room temperature. Total cholesterol was measured by fluorescence using the Amplex Red Cholesterol Assay Kit (Sigma-Aldrich Chemie, Steinheim, Germany), according to the manufacturer’s instructions [18]. For each sample, cholesterol concentration was normalized by the protein concentration of the total cell lysate, assessed by the microBCA assay (ThermoFisher Scientific, Waltham, MA, USA).

### 2.7. Statistical Analysis

Descriptive statistics were performed on all variables. Categorical variables were presented as absolute numbers and percentages. Continuous data were expressed as the mean  ±  standard deviation (SD) or as the median and interquartile range (Q1–Q3), as appropriate. Normality assumption was verified by graphical inspection. Baseline demographic, lifestyle, biochemical, and clinical characteristics were compared by BMI groups (normal weight: 18.5–24.99 kg/m^2^; overweight: 25–29.99 kg/m^2^; obesity: ≥30 kg/m^2^) for continuous variables with one-way analysis of variance (ANOVA) or Kruskal–Wallis test as appropriate. Categorical data were compared with the chi-square test or Fisher exact test as appropriate.

Univariate and multivariable linear regression models were used to test the relationship between NO production or macrophage cholesterol mass in HDL-treated cells and PM exposure as the continuous predictor. Multiple analysis was adjusted for a priori covariates (age, gender, BMI, smoking habits, and HDL cholesterol) and for variables that were significantly related to NO production or cholesterol mass in univariate analysis (*p*  <  0.05). To determine the best-performing models, we ran several regression equations separately for evaluating the β coefficients, standard errors (SE), *p*-values, as well as the goodness of fit (R2). Finally, the best models selected to predict the association between NO production or macrophage cholesterol mass and PM exposure were adjusted for: age, gender, BMI, smoking habits, HDL cholesterol, triglycerides, interleukin-8, and apparent temperature on the day of recruitment. MtDNAcn, an established biomarker of oxidative stress, was not associated with NO production and macrophage cholesterol mass.

To examine the potential effect modification of BMI, we added the interaction term PM * BMI to the multivariable selected models. We evaluated whether the effect of PM_10_ and PM_2.5_ exposure on NO production and macrophage cholesterol mass differs depending on BMI levels.

All statistical analyses were performed with SAS software (version 9.4; SAS Institute Inc., Cary, NC, USA). Two-sided *p*-values below 0.05 were considered statistically significant.

## 3. Results

### 3.1. Anthropometric, Biochemical, and Clinical Features of Enrolled Subjects

Enrolled subjects were mainly females and had a mean age of 52.1 ± 9.6 years (Table 1). On average, their lipid profile was characterized by normal plasma levels of HDL-C and triglycerides and by borderline levels of total and LDL cholesterol (Table 1). Subjects were stratified according to their BMI into three groups: BMI 18.5–24.9 kg/m^2^ (n = 23), BMI 25–29.9 kg/m^2^ (n = 26) and BMI ≥ 30 kg/m^2^ (n = 42) (Table 1). Subjects with higher BMI levels were older and characterized by lower levels of education and occupation. In addition, higher BMI levels were associated with higher triglycerides and lower HDL-C. Interestingly, no differences were detected in inflammatory and oxidative stress markers. The prevalence of diabetes was similar among the three groups, while the prevalence of hypertension significantly increased with BMI (Table 1).

The estimated levels of exposure to PM_10_ and PM_2.5_ in the last week before recruitment are reported in Table 2. A similarity in pollutant concentrations across the days of exposure was evident. Mean PM_10_ concentrations were above the annual regional air quality standards of 40 μg/m^3^. Mean PM_2.5_ concentrations were higher than the annual limits set at 25 μg/m^3^.

### 3.2. Production of NO by Endothelial Cells

**Figure 1:**
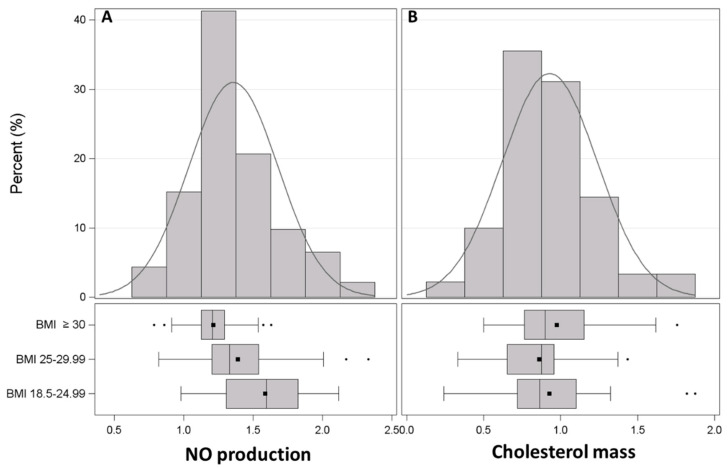
Frequency distribution of HDL function and impact of BMI. (**A**), Frequency distribution of NO production by endothelial cells exposed to apoB-D sera in the whole cohort and by BMI groups. Data are expressed as fold increase in untreated cells. (**B**), Frequency distribution of cholesterol mass of macrophages exposed to apoB-D sera in the whole cohort and by BMI groups. Results are expressed as fold reduction in untreated cells. All data are presented as histograms and boxplots; squares indicate the mean values and dots the outliers.

**Figure 2:**
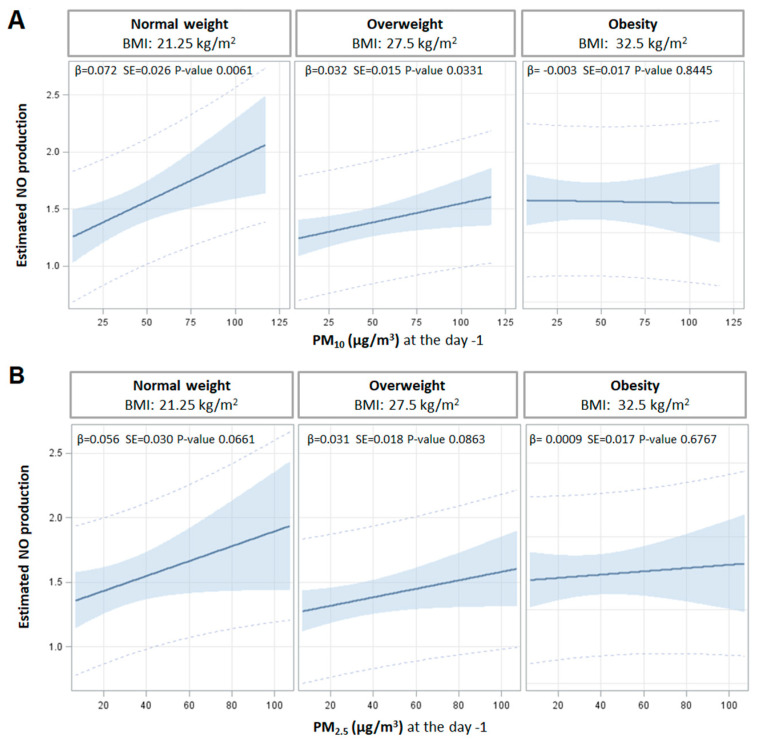
Interaction effect of particulate matter on NO production. Interaction effect of PM_10_ (**A**) and PM_2.5_ (**B**) and BMI on NO production by endothelial cells exposed to apoB-D sera. Strength of association between PM the day before the enrollment and NO production at three selected levels of BMI (normal weight 21.75 kg/m^2^, overweight 27.5 kg/m^2^, and obesity 32.5 kg/m^2^). Adjusted β regression coefficients were reported for 10 µg/m^3^ increases in PM concentration, at each level of BMI. *p*-values of interaction terms PM*BMI were 0.0203 and 0.2163, respectively, for PM_10_ and PM_2.5_. Linear regression models in both panels A and panel B were adjusted for age, gender, smoking habit, HDL-C, triglycerides, IL-8, and apparent temperature on the day of recruitment.

An average 1.36 ± 0.32-fold increase in NO production was observed in endothelial cells after treatment with HDL. When subjects were divided according to their BMI, HDL’s ability to promote NO release progressively declined with the increase in BMI (*p* < 0.0001, Figure 1A). Indeed, NO production was 1.59 ± 0.32-fold in the group with BMI 18.5–24.9 kg/m^2^ and decreased to 1.21 ± 0.20 fold in the group with BMI ≥ 30 kg/m^2^. No association was found between NO production and PM_10_ or PM_2.5_ exposures (Table 3). Nevertheless, when the interaction term between BMI and PM concentrations was taken into account, a significant modifying effect of BMI on PM_10_ was observed on the day before the recruitment. In subjects with a normal BMI, we found a positive association between day -1 PM_10_ exposure and NO production (Figure 2A): HDL’s ability to induce NO release increased by 0.072 fold every 10 μg/m^3^ increase in PM_10_ concentration measured at day -1 (*p* = 0.0061). In overweight subjects, NO production still increased by 0.032 fold every 10 μg/m^3^ increase in PM_10_ concentration measured at day -1 (*p* = 0.0331). On the contrary, HDL compensatory response to PM_10_ exposure was completely lost in participants with BMI ≥ 30 kg/m^2^, with a tendency towards a negative association (Figure 2A). Although not statistically significant, a similar interaction between BMI and PM_2.5_ was observed (Figure 2B).

### 3.3. Macrophage Cholesterol Mass

The exposure to HDL reduced cholesterol mass in THP-1-derived macrophages by 0.93 ± 0.31 fold. HDL’s ability to promote cell cholesterol removal was independent of BMI (*p* = 0.34, Figure 1B). No association was found between macrophage cholesterol mass after HDL and PM_10_ or PM_2.5_ exposures (Table 3).

BMI interacted with the association between cholesterol mass after HDL and PM_10_ exposure measured the day -1 (Table 4). Indeed, in subjects with normal BMI, a significant negative relationship between cell cholesterol mass after HDL and PM_10_ exposure was observed, with a 0.065-fold decrease every 10 μg/m^3^ increase in PM_10_ (*p* = 0.034). This association was markedly attenuated in overweight subjects and completely lost in subjects with BMI ≥ 30 kg/m^2^, with a tendency towards a positive association.

The modifying effect of BMI on the relationship between cell cholesterol mass after HDL and PM_2.5_ exposure was not significant, with the three groups for BMI categories displaying similar associations (Table 4).

## 4. Discussion

This retrospective analysis showed that HDL function, especially HDL’s endothelial protective activity, was affected by short-term exposure to air pollutants. More interestingly, the effect of PM exposure on HDL function was modulated by BMI. Indeed, in subjects with normal weight, a compensatory response was observed, i.e., HDL’s ability to induce NO production increased with exposure to higher concentrations of air pollutants the day before sample collection. However, this compensatory response was progressively lost at increasing BMI levels. Similarly, the ability of HDL to reduce cholesterol mass in macrophage-like cells increased with exposure to higher levels of PM only in subjects with normal weight. In addition, our results confirmed an impaired HDL ability to preserve endothelial homeostasis in obesity [7] and integrate previous findings showing impaired antioxidant and anti-inflammatory activities of HDL after PM exposure [19]. Exposure to environmental pollutants is known to trigger inflammation and oxidative stress and HDL should be able to limit the extent of such events. However, the loss of the compensatory response observed in subjects with elevated BMI likely indicates that in this condition, which is a chronic inflammatory status per se, HDL lose their ability to manage additional detrimental stimuli. Since inflammation and oxidative stress could directly impact HDL composition and function, related markers, such as interleukin 8 and mitochondrial DNA copy number, and smoking habits were included in the model. Similarly, the outdoor temperature was included, since it could influence the effect of PM on HDL function, as previously shown [20]. Finally, obesity alters plasma lipid profile, resulting in higher triglyceride and lower HDL-C levels in subjects with elevated BMI; thus, these variables were also included in the model.

Nitric oxide is a key player in the regulation of vascular tone and in preserving the endothelium as an anti-adhesive surface for circulating cells [21]. Consequently, a reduction in its availability is associated with impaired vasodilation, as previously observed in many clinical conditions. Rapid alterations in systemic microvascular and macrovascular tone were also observed after short-term exposure to coarse, fine, and ultrafine PM [9,22,23]. With HDL being the main activator of endothelial NO synthase (eNOS) in serum [17], an impairment of its function could explain the reduced vasodilation after PM exposure, together with sympathetic nervous system activation [24]. Interestingly, the significant modifying effect of BMI on the interaction between HDL function and PM exposure is consistent with the higher impairment of vasodilation observed in subjects with obesity [25]. Thus, our results provide additional evidence supporting endothelial dysfunction as a key mechanism leading to ischemic events in subjects exposed to environmental pollutants, especially in those with obesity [26].

Similar results were obtained when another HDL atheroprotective function was assessed, i.e., the ability to promote the removal of cholesterol from peripheral cells, including macrophages in the arterial wall. In this case, HDL’s ability to reduce cholesterol mass was not affected by BMI and the modifying effect of BMI on the interaction between HDL functionality and PM exposure was attenuated if compared to that observed for NO production. Previous studies assessed the impact of environmental pollutants on cholesterol efflux to HDL with variable results [8]. These inconsistencies could be partly explained by the different cell types and experimental settings used, which can be specific for one or some efflux pathways. Thus, in the present study, we decided to evaluate the net changes in cholesterol mass in LDL-loaded macrophages after HDL exposure in order to provide an overall estimation of HDL effect, irrespective of the specific underlying efflux mechanisms.

In the literature, there is evidence of an association of both short- and long-term exposures to environmental pollutants with cardiovascular morbidity and mortality [27]. Long-term exposure could favor the development and progression of atherosclerosis by establishing a chronic low-grade inflammatory state and by upregulating the sympathetic nervous system [3]. Regarding the clinical relevance of short-term exposure, the transient loss of a compensatory response by HDL that we observed in subjects with elevated BMI supports the hypothesis that a susceptible individual, who is at high risk for IHD due to multiple risk factors, may experience an event or face an unfavorable prognosis when acutely exposed to high levels of PM.

The present study has some limitations. PM exposure was estimated according to air-quality-monitoring-station data, whose measurements might inaccurately capture the real exposure at residential addresses. The exposure assessment did not include personal monitoring; thus, we were not able to take into account the indoor activities of the subjects. Obesity is a clinical condition characterized by several chronic comorbidities. Thus, although we evaluated the possible contribution of these diseases in modulating HDL function, we cannot completely exclude they are part of the “obesity” effect rather than the BMI only. In addition, we cannot rule out the effect of eNOS gene polymorphisms on the relationship between HDL function and PM exposure, since these DNA variants were previously shown to modify the effect of PM on oxidative stress biomarkers [28]. Even if blood samples were collected, processed, and stored according to standardized procedures, we cannot completely exclude that storage time could have partially affected HDL function. Lastly, the limited sample size and the observational design of the study highlight an association between HDL function and PM exposure but foreclose any causative link.

## 5. Conclusions

Our results support the hypothesis of a transient impairment of NO-mediated vasodilation after PM exposure [9] and suggest a role for HDL dysfunction among the underlying mechanisms. Moreover, the loss of the compensatory response of HDL function to PM exposure at increasing BMI levels could help to explain why subjects with obesity seem more susceptible to the detrimental effects of air pollution.

## Figures and Tables

**Table 1:** Anthropometric, biochemical, and clinical features of enrolled subjects.

	All	BMI 18.5–24.9	BMI 25–29.9	BMI ≥ 30	*p*-Value
	(n = 91)	(n = 23)	(n = 26)	(n = 42)	
Age, years	52.1 ± 9.6	45.0 ± 5.6	52.3 ± 8.8	55.6 ± 9.8	<0.0001
Gender					
Males	27 (29.67%)	4 (17.39%)	13 (50.00%)	10 (23.81%)	0.0306
Females	64 (70.33%)	19 (82.61%)	13 (50.00%)	32 (76.19%)	
BMI, kg/m^2^	28.9 ± 5.0	21.8 ± 1.9	28.0 ± 1.6	33.3 ± 1.6	-
Smoking status					
Never smoker	49 (53.85%)	17 (73.91%)	11 (42.31%)	21 (50.00%)	0.0739
Former smoker	26 (28.57%)	3 (13.04%)	12 (46.15%)	11 (26.19%)	
Current smoker	16 (17.58%)	3 (13.04%)	3 (11.54%)	10 (23.81%)	
Education					
Primary school	3 (3.30%)	0 (0.0%)	1 (3.85%)	2 (4.76%)	<0.0001
Secondary school	16 (17.58%)	0 (0.0%)	2 (7.69%)	14 (33.33%)	
High school	35 (38.46%)	5 (21.74%)	9 (34.62%)	21 (50.00%)	
University or more	37 (40.66%)	18 (78.26%)	14 (53.85%)	5 (11.9%)	
Occupation					
Employee	72 (79.12%)	23 (100.0%)	21 (80.77%)	28 (66.67%)	0.0189
Unemployed	0 (0.0%)	0 (0.0%)	0 (0.0%)	0 (0.0%)	
Pensioner	14 (15.38%)	0 (0.0%)	4 (15.38%)	10 (23.81%)	
Housewife	5 (5.49%)	0 (0.0%)	1 (3.85%)	4 (9.52%)	
Total cholesterol, mg/dL	212.6 ± 43.2	213.8 ± 33.1	211.7 ± 44.4	213.0 ± 47.6	0.9368
HDL cholesterol, mg/dL	56.9 ± 17.6	68.0 ± 16.5	59.0 ± 16.3	49.6 ± 15.6	0.0001
LDL cholesterol, mg/dL	133.3 ± 36.9	126.3 ± 27	129.7 ± 34.8	139.9 ± 41.7	0.2664
Triglycerides, mg/dL	113 [77;162]	83.2 [69.7;116.1]	93.4 [73;142.7]	145.5 [109;215]	0.0001
Interleukin-8, pg/mL	6.7 ± 6.83	7.6 ± 6.1	8.4 ± 11.9	5.1 ± 4.8	0.2500
N. of subjects with IL-8 < LLOQ	25 (27.47%)	0 (0.0%)	5 (19.23%)	20 (48.78%)	-
Mitochondrial DNA, cn	1.08 ± 0.31	1.00 ± 0.23	1.13 ± 0.39	1.08 ± 0.29	0.3358
Diabetes					
Yes	9 (9.89%)	0 (0.0%)	2 (7.69%)	7 (16.67%)	0.0930
No	82 (90.11%)	23 (100.0%)	24 (92.31%)	35 (83.33%)	
Hypertension					
Yes	40 (43.96%)	1 (4.35%)	9 (34.62%)	30 (71.43%)	<0.0001
No	51 (56.04%)	22 (95.65%)	17 (65.38%)	12 (28.57%)	

Enrolled subjects (n = 91) were divided into three groups according to BMI categories. For normal distribution, values are expressed as mean ± SD and one-way ANOVA was applied. When not normally distributed, values are expressed as median [Q1;Q3] and Kruskal–Wallis test was used. For categorical variables, values are reported as frequencies and percentages and chi-square test or Fisher exact test was applied, as appropriate. LLOQ, lower limit of quantification; cn, copy number.

**Table 2:** Mean concentrations of PM_10_ and PM_2.5_ exposure evaluated from one week before the enrollment of participants.

	Mean ± SD
**PM_10_ exposure (µg/m^3^)**	
Day 0	50.61 ± 22.49
Day -1	48.35 ± 22.01
Day -2	47.36 ± 22.02
Day -3	51.61 ± 27.68
Day -4	50.67 ± 24.00
Day -5	53.09 ± 22.97
Day -6	49.72 ± 24.41
**PM_2.5_ exposure (µg/m^3^)**	
Day 0	38.19 ± 20.82
Day -1	36.23 ± 18.47
Day -2	37.97 ± 19.49
Day -3	34.37 ± 17.93
Day -4	37.51 ± 17.38
Day -5	41.22 ± 18.59
Day -6	38.92 ± 20.58
**Apparent temperature (°C)** Day 0	7.37 ± 4.81

PM, particulate matter.

**Table 3:** Association between HDL-mediated NO production, HDL-mediated reduction in cholesterol mass, and PM exposures.

	NO Production			Cholesterol Mass		
	β	SE	*p*-Value	β	SE	*p*-Value
**PM_10_ exposure**						
Day 0	0.016	0.014	0.2757	−0.014	0.016	0.3909
Day -1	0.021	0.014	0.1411	−0.016	0.016	0.3320
Day -2	0.003	0.014	0.8424	0.004	0.016	0.8103
Day -3	−0.002	0.011	0.8590	0.011	0.012	0.3721
Day -4	−0.004	0.013	0.7667	0.021	0.014	0.1421
Day -5	0.004	0.013	0.7536	0.009	0.015	0.5560
Day -6	0.006	0.013	0.6399	0.007	0.014	0.6001
**PM_2.5_ exposure**						
Day 0	0.007	0.015	0.6322	−0.027	0.017	0.1082
Day -1	0.025	0.017	0.1468	−0.034	0.020	0.0916
Day -2	0.020	0.016	0.2119	−0.008	0.018	0.6843
Day -3	0.008	0.017	0.6329	−0.005	0.019	0.8068
Day -4	0.008	0.018	0.6726	0.002	0.020	0.9254
Day -5	0.008	0.018	0.6672	−0.011	0.020	0.5759
Day -6	0.030	0.017	0.0815	−0.002	0.020	0.9037

β regression coefficients represent the increase in NO production/cell cholesterol mass for 10 μg/m^3^ increase in PM_10_ or PM_2.5_ concentration. Linear regression models were adjusted for age, gender, BMI, smoking habits, HDL-C, triglycerides, interleukin-8 and apparent temperature on the day of recruitment.

**Table 4:** Modifying effect of BMI on the association between HDL-mediated reduction in macrophage cholesterol mass and PM exposure the day before the enrollment.

Independent Variable	BMI	β	SE	*p*-Value	*p*-Value for Interaction
**PM_10_ Day -1**	**Normal weight: 21.25 kg/m^2^**	−0.0650	0.0301	0.0342	0.0583
	Overweight: 27.5 kg/m^2^	−0.0270	0.0170	0.1191	
	Obesity: 32.5 kg/m^2^	0.0067	0.0197	0.7365	
**PM_2.5_ Day -1**	Normal weight: 21.25 kg/m^2^	−0.0560	0.0368	0.1309	0.4724
	Overweight: 27.5 kg/m^2^	−0.0390	0.0211	0.0687	
	Obesity: 32.5 kg/m^2^	−0.0240	0.0241	0.3211	

Strength of association between PM the day before the enrollment and cholesterol mass at three selected values of BMI. Adjusted β regression coefficients were reported for 10 µg/m^3^ increases in PM concentration, at each level of BMI. Linear regression models were adjusted for age, gender, smoking habit, HDL-C, triglycerides, IL-8, and apparent temperature on the day of recruitment.

## Data Availability

The data presented in this study are available on request from the corresponding authors. The data are not publicly available due to ethical reasons.
